# The Influence of Emotional State on the Masticatory Muscles Function in the Group of Young Healthy Adults

**DOI:** 10.1155/2015/174013

**Published:** 2015-03-26

**Authors:** Stocka Anna, Kuc Joanna, Sierpinska Teresa, Golebiewska Maria, Wieczorek Aneta

**Affiliations:** ^1^Department of Prosthetic Dentistry, Medical University of Białystok, Sklodowska-Curie Street 24a, 15-089 Białystok, Poland; ^2^Department of Prosthetic Dentistry, Jagiellonian University Medical College, Montelupi Street 4, 31-155 Cracow, Poland

## Abstract

Stress may affect the function of all the components of the masticatory system and may ultimately lead to differentiated symptoms and finally to systemic and structural dysfunctions. *Objective.* To determine the effect of stress on the masticatory muscles function in young healthy adults. *Material and Methods*. A total of 201 young, Angle's first class, healthy volunteers, 103 female and 98 male, in the age between 18 and 21 years were recruited into the study. All the participants underwent clinical examination according to the Slavicek scheme, questionnaire survey according to Perceived Stress Scale, and assessment of masticatory muscles function in central occlusion. *Results*. Symptoms of masticatory system dysfunction were found in the group of 86 subjects (46,24%). All the muscles activity in central occlusion was comparable in female and male groups. Mean values of masseters activities in the group of low stress subjects (75,52 *µ*V ± 15,97) were statistically different from the groups with medium (82,43 *µ*V ± 15,04) and high (81,33 ± 12,05) perceived stress (*P* < 0.05). *Conclusion*. Chronic stress may reveal or exacerbate symptoms of masticatory dysfunction.

## 1. Introduction

A number of stress-related disorders (SRDs) may develop or exacerbate due to chronic stress. The disorders include obesity, type 2 diabetes, atherosclerosis, idiopathic hypertension, cardiac ischemia, peptic ulceration, irritable bowel syndrome, headaches, neck pain, migraine, spine aches, osteoporosis, and dermatological complications. The most common stress-related symptoms affecting mental health can vary from sleep disorders, depressed mood, sadness, restlessness, irritability, anxiety, and impaired concentration and memory to chronic fatigue syndrome, traumatic stress, social stress, anorexia nervosa, or bulimia. Stress is believed to underlie first episodes of depression, which in most people appear in adolescence or early adulthood. According to many researchers, stress also affects the function of the masticatory system [1–7]. Any surpassing of the adaptive potential of the body may lead to pathological reactions, especially in high-energy or low-resistance tissues. Dental arches are the somatic sites where excessive psychoemotional tension can be diffused and reduced [8–11]. This is manifested by clenching or grinding the teeth (bruxism). If the psychoemotional tension persists, strain and/or ischemia may appear in the overloaded muscles and trigger points are activated, causing myofascial pain and its sequels. All components of the masticatory system may be affected, which ultimately leads to differentiated symptoms, from the systemic (headaches, neck pain, shoulder girdle pain, and backache) to structural dysfunctions, such as hypertrophy and tenderness of masseter muscle attachments or increased tension in these muscles. Hard dental tissues show attrition, worn teeth, cervical lesions, periodontal atrophy, excessive dental mobility, pain of unknown etiology, anemisation of lingual and buccal mucosa, and temporomandibular joint (TMJ) disorder symptoms: cracking, clicking, reduced mobility, and pain on palpation [9].

According to the International Headache Society (IHS) criteria and guidelines of American Academy of Orofacial Pain (AAOP) [12], masticatory dysfunction can be diagnosed when at least three of the following symptoms are reported: pain and acoustic signs on mandibular movement, limited mandibular movement, difficulty in mouth opening, and occlusal or nonocclusal parafunctions. The etiology of masticatory dysfunction is diverse. Apart from genetic and environmental determinants, also a psychogenic factor may be responsible, including civilization-related stress that reduces adaptive potentials of the human body [1, 3, 13, 14]. Anxiety, restlessness, and depression states may induce or exacerbate masticatory dysfunction. A number of clinical studies seem to confirm the relationship between the exacerbation of masticatory dysfunction and strong emotional experiences [4, 6, 15–19], especially in young individuals in the final stage of maturation and early adulthood (18–21 years). It is extremely important that the affected people should be immediately diagnosed and treated.

The study objective was to determine the effect of stress on the function of the masticatory muscles in young healthy adults.

## 2. Material

A total of 201 young people were randomly chosen, 103 female and 98 male aged 18–21 years (mean 19 years).

Inclusion in the study required participants to satisfy the following criteria:

1. class I molar and canine relations;
2. full natural dentition with well-aligned arches;
3. vertical, transverse, and anteroposterior relationships well-related;
4. normal growth and good health.

Subjects were excluded from the study when they demonstrated the following:

1. previous orthodontic treatment;
2. extensive fillings or edentulous spaces;
3. history of trauma in the region of masticatory system;
4. any treatment concerning pain in any region of masticatory system;
5. prosthetic treatment before recruitment to the study.

The data were collected in the Department of Prosthodontics at the Medical University of Białystok, Poland, and the protocol conformed to the criteria of The Helsinki Declaration, ICH Guideline for Good Clinical Practice, and approved by the Ethical Committee of Jagiellonian University, Poland, with an approval number of KBET/89B/2009. The participants were recruited into the study after obtaining consent from educational authorities, school headmasters, parents, and participants themselves.

## 3. Methods

The study included the following:

1. clinical examination according to the Slavicek scheme [20];
2. questionnaire survey (Perceived Stress Scale PSS-10);
3. assessment of the masticatory muscles function (electromyography of four pairs of masticatory muscles (EMG) in central occlusion).

### 3.1. Clinical Examination according to the Slavicek Scheme

All study participants underwent clinical examination according to the Slavicek scheme [20], which consists of general medical history, specialistic history, and clinical examination. In the first part, the focused history was taken on general health condition, that is, past or present infections, allergies, hormonal disorders, or psychological problems. The second part concerned dental complaints, especially problems during mastication, speaking, hypersensitivity of the teeth, pain or acoustic phenomena in TMJ during mouth opening, biting, and yawning, headaches, and bad postures. Other questions referred to the past history of serious injury, accident, or intubation. Then, the study participant was asked about past orthodontic therapy and treatment with occlusal splints. Final questions concerned mental state of the respondent, subjective opinion of the complaint severity, and the need for treatment.

Clinical examinations of the muscles, TMJ, and facial nerves were performed. Pain and excessive tension or hypertrophy were assessed by palpation. Intraoral and extraoral examinations included checking muscles of the head, neck, shoulders, temples, and masseters, pterygoid muscles, mylohyoid muscles, digastric muscles, suprahyoid and subhyoid muscles, sternocleidomastoid muscles, maxillary tumors, tongue, larynx, and base of the temporomandibular ligament.

The TMJ was examined by simultaneous palpation in statics and rotation and by checking the acoustic symptoms during lowering, lifting, and lateral movements of the mandible. Function of facial nerves was assessed. Olfactory nerve, optic nerve, oculomotor nerve, compound nerve, trigeminal nerve, abducens nerve, facial nerve, vestibulocochlear nerve, linguopharyngeal nerve, vagus nerve, accessory nerve, and sublingual nerve were examined.

One well-qualified researcher performed all of the clinical examinations to avoid potential disagreement. For reliability, another investigator, who was also familiar with the method used, repeated the clinical examination for 10% of the participants. Ninety percent agreement was found.

### 3.2. Questionnaire Survey

A questionnaire form was used, that is, a psychological scale designed by Cohen et al., the so-called Perceived Stress Scale (PSS-10). The scale contains 10 questions concerning various subjective feelings associated with problems and personal events that have occurred during the last month. There is a short introductory set of instructions at the top of the questionnaire. The respondents answered the questions using a five-grade scale (0–4), with 0 meaning never, 1 hardly ever, 2 sometimes, 3 quite often, and 4 very often. Calculation of the overall intensity of perceived stress followed a change in the score in positively formulated answers, that is, according to the rule, 0 = 4, 1 = 3, 3 = 1, and 4 = 0. The overall score obtained after summing up all the answers was then converted into standard units (stens). The higher the score, the greater the intensity of perceived stress and thus the lower the resistance to it (Table 1).

### 3.3. Assessment of the Masticatory Muscles Function

The EMG examination was performed using an EMG apparatus (BioResearch, Inc., Milwaukee, WI, USA). Electric potentials of four pairs of muscles were measured: anterior parts of temporal muscles, masseters, anterior bellies of digastric muscles, and sternocleidomastoid muscles. The muscular activity was registered using bipolar adhesion electrodes at the maximum intercuspal position (central occlusion). Bipolar electrodes covered with silver chloride (Ag/AgCl), with constant distance between the poles (19 mm between the centers of poles), were placed on the surface of the skin degreased with alcohol, over the most bulged muscle mass palpated in contraction, parallel to muscular fibers. The zero electrode was placed in the supraclavicular pit [21]. Before EMG registration, all the subjects were instructed in the procedure. They were instructed to hold their teeth firmly together for 1-2 seconds and then open and repeat the maximum clench 2 more times. Once 3 clenches were recorded, subsequently, they were instructed to perform lateral excursive movements, separately left lateral and right lateral. Each measurement was performed three times with a minute break between subsequent registrations. The mean of three measurements was calculated for analysis.

### 3.4. Statistical Analysis

The study parameters were divided according to qualitative and quantitative features. Bipartite tables with calculated percentage distributions were applied for the qualitative features. For the quantitative features, average and dispersion measures were used, that is, the arithmetic mean and standard deviation. An analysis of variance (ANOVA) was used to determine if the differences in the parameters analyzed between the groups were significant. The strength of relationships between pairs of measurable parameters was determined using Pearson's correlation coefficient or Spearman's correlation rank coefficient, and its significance was assessed using Student's *t*-test to evaluate the correlation coefficient. Differences in the correlation were considered significant at *P* < 0.05.

Statistical analysis was conducted using the STATISTICA 8.0.PL software.

## 4. Results

The medical history and its clinical verification revealed masticatory system dysfunction in 86 generally healthy subjects (46%), showing no masticatory complaints during recruitment into the study. The major complaints reported by the study subjects with diagnosed masticatory dysfunction included headaches, pain on wide mouth opening, and muscle pain. The study participants had no history of general complaints or previous medical interventions (Table 2).

The PSS-10 is a method designed to examine stress and ways of stress management. According to the scale score value, the study group was divided into three subgroups as follows:

- group I, 1–4 stens (0–13 points), low score,
- group II, 5-6 stens (14–19 points), medium score,
- group III, 7–10 stens (20–40 points), high score.

The lowest scores in the PSS were noted in approximately 29% of the study subjects, medium scores in nearly 40%, and the highest scores in almost 31%. In group I (the lowest PSS score), the number of women was smaller as compared to that of men. However, in group II, the number of women and the number of men were comparable. The highest number of women was noted in group III, the number of men in this group being more than twice lower (Table 3).

The distribution of masticatory dysfunction symptoms in the respective groups is presented in Table 4. The percentages of study participants with early symptoms of dysfunction in the respective groups versus all patients in these groups were comparable. Among study subjects with diagnosed early symptoms of masticatory dysfunction high level of perceived stress was observed in 24 people, accounting for 28% of this group.

The EMG examination was performed to assess four pairs of masticatory muscles: anterior temporal muscles, masseters, anterior bellies of the digastric muscles, and sternocleidomastoid muscles. The levels of activity of all muscles in central occlusion were comparable in female and male groups. Higher activity was noted on the right side in the masseters in men, with the mean values of electric potentials being significantly higher than in their female counterparts. In male group, the sum of mean values of electric potentials in central occlusion of temporal muscles and masseters on the right and left side was higher and statistically significantly different than in female group (Table 5).

The assessment of the activity of muscles in the PSS-based groups revealed a significant difference between the mean value of electric potentials of masseters and the sum of electric potentials of temporal muscles and masseters in central occlusion in the respective groups. The mean values of electric potentials of masseters in group I were considerably lower than in group II and group III. The sum of the mean values of electric potentials of temporal muscles and masseters on the right was the highest in group III and differed significantly as compared to group I and group II. No significant differences were found between the mean values of electric potentials for the other muscles in groups I, II, and III (Table 6).

## 5. Discussion

Numerous clinical studies prove that stress and psychological state of a patient have a substantial effect on the activity of the masticatory system. In the masticatory organ, chronic stressful situations trigger excessive muscular tension which is frequently reduced by motor reactions, parafunctions. The reactions may lead to masticatory system dysfunction [3, 22–29]. TMJ dysfunctions are considered the third most common disorder of the masticatory system, after caries and periodontal diseases [13, 30]. All age groups can be affected with various intensity, with the prevalence rate of 60–80%, even up to 93% of the human population [31–33]; however, only 3–7% require treatment [25].

Epidemiological studies prove that increased intensity of TMJ dysfunction can be observed between the age of 20 and 40 years, that is, in young and middle age adults, more commonly in women than men [22, 23, 25]. Genetic predisposition and hormonal factor may play an essential role, especially in puberty, reproductive period, and menopause characterized by hypersensitivity of women to psychosocial factors and everyday stress [3, 24, 26–28]. Our own study showed that in the high stress group the number of women was twice as high as men and that symptoms of masticatory dysfunction were observed in 46.24% of the whole study group. According to literature data, among the key factors in the etiopathogenesis of the stomatognathic disorders, excessive muscle tension and chronic stress account for 34% and mental diseases for 7.1% [10].

Based on the medical history and clinical verification in the current study, the percentage of subjects with symptoms of masticatory dysfunctions in the PSS groups as compared to the overall group was 13.87% in group I, 19.71% in group II, and 12.08% in group III. Among subjects with symptoms of disturbed function of the masticatory system, 27.9% had a high level of perceived stress. The higher number of people with masticatory dysfunction symptoms in group II (medium level of stress) may be due to the fact that it was the most numerous group. However, the slightly higher percentage of patients with symptoms of masticatory dysfunction in group I as compared to group III may prove that stress is only one of many etiological factors that disturb masticatory function.

In the current study, the effect of stress on the activity of muscles was assessed by measuring the activity of masseters and anterior temporal muscles in central occlusion in three PSS-dependent groups. The mean values of electric potentials of masseters in group I, that is, the lowest level of perceived stress, were lower than in group III, that is, with high level of felt stress, the differences being statistically significant (*P* < 0.05). In many patients, stress-induced tension is relieved through involuntary motor activities of the dental arches, resulting in parafunctions. The most common parafunction is bruxism. According to literature data, the majority of human populations (85–90%) at certain time of life clench or grind their teeth. Two types of bruxism have been distinguished: sleep bruxism (SB) and awake bruxism (AB). Lobbezoo et al. have shown that these two types differ in etiology, course, and effects [9]. SB occurs mainly during microawakening during sleep, unconsciously, and is manifested by tooth grinding. According to various studies, sleep episodes of bruxism occur in 5% to 8% of the world population [34, 35]. On the other hand, awake bruxism is a conscious patient-controlled phenomenon, manifested by soundless clenching of the teeth and lack of grinding, typical of SB. Its prevalence is estimated at approximately 20% of the general population. Awake bruxism is psychologically related and its intensity may depend on the emotional state of patients [36, 37]. AB can be an independent phenomenon or may accompany sleep bruxism. Until now, bruxism in dentistry has been defined as a parafunction, that is, unconscious abnormal fixed activity of the masticatory organ, differing in quality and quantity from the normal function. The latest results show that bruxism can be referred to as not a disorder but a physiological activity in response to stress [38], which was phylogenetically encoded in humans, where baring the teeth in primitive people meant warning [10].

The appearance or exacerbation of symptoms of masticatory dysfunction to a large extent depends on individual predisposition and the related specific features of character. Various personality tests are used to evaluate psychoemotional state of patients with stomatognathic system disorders. Mongini using the Minnesota Multiphasic Personality Inventory (MMPI) test [39] has found that approximately 40% of patients with masticatory system dysfunction show elevated levels of hysteria and hypochondria. These patients frequently complain of headaches and psychoemotional symptoms, such as anxiety, restlessness, and fear. Higher levels of psychological, behavioral, and somatic symptoms of stress in people with masticatory system dysfunctions as compared to healthy subjects have been confirmed by other authors [4, 18, 40–45]. The role of psychological factor, especially stress, neuroticism, and depression, in the etiology of functional disorders of the masticatory organ has been more frequently emphasized in patients with myofascial pain than in subjects with TMJ disorders [15–17, 19, 46]. Pain is a significant symptom of masticatory dysfunction, forcing patients to see a specialist. Thus, one of the latest classifications of functional disorders of the masticatory system, the Research Diagnostic Criteria for Temporomandibular Disorders (RDC/TMD) presented by Dworkin and LeResche in 1992, takes into account both the clinical factor of masticatory dysfunction and the psychological one, that is, stress, nervousness, depression, and somatization of pain [21, 47]. The classification, based on the general biopsychosocial model, is the best available diagnostic criterion of functional disorders of the masticatory system. It is recommended for scientific research and routine clinical examinations [48, 49]. Although stress and mental diseases are not the only factors of masticatory organ dysfunctions, they have an increasingly important role, especially in more vulnerable subjects. Therefore, detailed psychological diagnostic examinations, as well as psychosocial and psychiatric assessment, are recommended in patients with masticatory organ dysfunctions, who complain of chronic pain accompanied by anxiety, stress, or depression.

## 6. Conclusions

1. Among young healthy adults with a complete natural dentition, some individuals do not report symptoms of masticatory system dysfunction despite their presence. Those who complain of dysfunction symptoms do not associate them with the masticatory system.
2. Adults with higher level of perceived stress tended to have increased activity of masseters in central occlusion.
3. Chronic stress may reveal or exacerbate symptoms of masticatory dysfunction.

## Figures and Tables

**Table 1:** The intensity of perceived stress according to a psychological scale named Perceived Stress Scale (PSS-10).

Sten	PSS	Intensity of perceived stress
1–4 stens	0–13 points	Low
5-6 stens	14–19 points	Medium
7–10 stens	20–40 points	High

**Table 2:** The most common complaints in the group of subjects with masticatory system dysfunction symptoms.

Symptom	Number	%
Headache	42	49.00
Muscle pain	25	28.91
Pain during wide mouth opening, biting, or yawing	28	32.57
Acoustic phenomena in TMJ	22	26.40
Pain in the TMJ region	17	20.10
Pain in the region of head, neck, or nape	14	16.33
Clenching or grinding	19	22.06
Teeth sensitivity	18	21.35

**Table 3:** The number of men and women in the subgroups separated on the base of PSS-10 values.

PSS-10	Total		Male group		Female group	
	Number	%	Number	%	Number	%
Group I (low stress)	55	30%	34	18%	21	11%
Group II (medium stress)	74	40%	36	19%	38	20%
Group III (high stress)	57	30%	18	10%	39	21%

**Table 4:** The distribution of masticatory dysfunction symptoms in the respective groups separated on the base of PSS-10.

	Group I (low stress)			Group II (medium stress)			Group III (high stress)		
	Number	Total %	% in the group	Number	Total %	% in the group	Number	Total %	% in the group
Lack of dysfunction symptoms	29	16	53	38	21	51	33	18	58
Presence of dysfunction symptoms	26	14	47	36	20	49	24	12	42

**Table 5:** The levels of activity of all muscles (*µ*V) in central occlusion in female and male groups.

	Female group	Male group
	Mean ± SD	Mean ± SD
Temporales anteriores	72.25 ± 13.68	74.39 ± 15.99
Masseters	99.25 ± 13.86	120.45 ± 15.61
Sternocleidomastoideus	8.7 ± 7.4	8.6 ± 6.4
Digastricus	16.5 ± 15.0	16.8 ± 14.5

Symetry		
Temporal anterior right	72.91 ± 40.80	74.74 ± 37.06
Temporal anterior left	71.82 ± 40.24	77.26 ± 44.70
Masseter right	105.79^*^ ± 55.46	127.33^*^ ± 60.39
Masseter left	92.68^*^ ± 44.81	117.71^*^ ± 59.78

Synergy		
Anterior temporal muscle and masseter right	84.92^*^ ± 18.32	106.78^*^ ± 20.74
Anterior temporal muscle and masseter left	81.93^*^ ± 17.92	89.88^*^ ± 20.51

^*^Statistical difference between female and male groups (*P* < 0.05).

**Table 6:** The assessment of the muscles activities in the PSS-10-based groups (*µ*V) in central occlusion.

	Group I: PSS-10, low	Group II: PSS-10, medium	Group III: PSS-10, high
	Mean ± SD	Mean ± SD	Mean ± SD
Temporal muscle right	67.49 ± 18.93	75.19 ± 25.34	77.51 ± 29.87
Temporal muscle left	71.49 ± 20.60	74.09 ± 23.90	77.32 ± 23.08
Masseter right	120.25 ± 26.47	122.53 ± 23.43	103.37 ± 22.83
Masseter left	107.24 ± 31.16	108.22 ± 33.69	96.97 ± 36.07
Temporales muscles	69.20 ± 17.82	74.43 ± 11.34	77.66 ± 15.64
Masseters	110.52^∗x^ ± 15.97	115.23^*^ ± 15.04	100.33^x^ ± 12.05
Temporal muscle and masseter right (synergy)	93.54^x^ ± 20.39	98.86^i^ ± 17.84	90.44^xi^ ± 20.08
Temporal muscle and masseter left (synergy)	89.36 ± 21.82	91.15 ± 19.19	87.14 ± 17.63

^*^Statistical difference between groups I and II.

^x^Statistical difference between groups I and III.

^i^Statistical difference between groups II and III.
